# Influence of the Degree of Unsaturation in Fish Oil Supplements on Oxidative Stress and Protein Carbonylation in the Cerebral Cortex and Cerebellum of Healthy Rats

**DOI:** 10.3390/antiox13111408

**Published:** 2024-11-17

**Authors:** Francisco Moreno, Lucía Méndez, Ingrid Fernández, Bernat Miralles-Pérez, Montserrat Giralt, Marta Romeu, Sara Ramos-Romero, Josep Lluís Torres, Isabel Medina

**Affiliations:** 1 Institute of Marine Research—Spanish National Research Council (IIM-CSIC), Eduardo Cabello 6, 36208 Vigo, Spain; fmoreno@iim.csic.es (F.M.); ifernandez@iim.csic.es (I.F.); bmiralles@iim.csic.es (B.M.-P.); medina@iim.csic.es (I.M.); 2 University of Vigo, Circunvalación ao Campus Universitario, 36310 Vigo, Spain; 3 Pharmacology Unit, Faculty of Medicine and Health Sciences, Universitat Rovira i Virgili, Sant Llorenç 21, 43201 Reus, Spain; montse.giralt@urv.cat (M.G.); marta.romeu@urv.cat (M.R.); 4 Department of Cell Biology, Physiology and Immunology, Faculty of Biology, University of Barcelona, Av. Diagonal 643, 08028 Barcelona, Spain; sara.ramosromero@ub.edu; 5 Nutrition & Food Safety Research Institute (INSA-UB), Maria de Maeztu Unit of Excellence, Prat de la Riba 171, 08921 Santa Coloma de Gramenet, Spain; joseplluis.torres@iqac.csic.es; 6 Institute of Advanced Chemistry of Catalonia—Spanish National Research Council (IQAC-CSIC), Jordi Girona 18-26, 08034 Barcelona, Spain

**Keywords:** fish oil, soybean oil, coconut oil, oxidative damage, endogenous antioxidant defenses, brain

## Abstract

ω-3 polyunsaturated fatty acids (PUFAs) are crucial for brain structure and function, especially docosahexaenoic acid (DHA). However, an excess of DHA may increase lipid peroxidation due to its high degree of unsaturation, particularly in tissues highly susceptible to oxidative stress, such as the brain. Therefore, this study evaluated the effects of 10 weeks of dietary supplementation with fish oil containing 80% DHA on oxidative stress and the modulation of the carbonylated proteome in both the cerebral cortex and cerebellum of male Sprague Dawley rats. The results were compared with those induced by oils with a lower degree of fat unsaturation (fish oil containing 25% DHA and 25% eicosapentaenoic acid, soybean oil containing 50% linoleic acid and coconut oil containing 90% saturated fat). The results demonstrated that fish oil containing 80% DHA significantly increased the ω3/ω6 ratio in both the cortex and cerebellum while stimulating antioxidant defense by enhancing the reduced glutathione amount and decreasing the carbonylation of specific proteins, mainly those involved in glycolysis and neurotransmission. The majority of sensitive proteins in both brain regions followed this carbonylation trend (in decreasing order): soybean > EPA/DHA 1:1 > coconut > 80% DHA. The results also indicated that the cerebellum is more responsive than the cortex to changes in the cellular redox environment induced by varying degrees of fat unsaturation. In conclusion, under healthy conditions, dietary supplementation with fish oils containing high DHA levels makes the brain more resilient to potential oxidative insults compared to oils with lower DHA content and a lower degree of fatty acid unsaturation.

## 1. Introduction

The consumption of long-chain ω-3 polyunsaturated fatty acids (PUFAs), primarily sourced from fish and seafood in human diet, has been linked to a variety of health benefits that involve many bodily systems and the prevention and palliation of several conditions, including cardiovascular diseases and type 2 diabetes [1].

Recent epidemiological studies and clinical trials have indicated that increasing the dietary intake or nutritional supplementation of ω-3 PUFAs, especially eicosapentaenoic acid (EPA) and docosahexaenoic acid (DHA), is closely associated with a reduced risk or therapeutic benefits for developing cognitive disorders and neurodegenerative diseases [2]. This is because ω-3 PUFAs are particularly relevant for the central nervous system (CNS). The CNS is notably enriched in these ω-3 PUFAs, especially DHA, which serves as a crucial structural component for maintaining cellular functional integrity [3].

ω-3 PUFAs, along with ω-6 PUFAs, are essential for vertebrates, as they cannot be synthesized de novo in sufficient quantities and must be obtained through diet [4]. Consequently, the fatty acid composition of the diet significantly impacts brain health. This dietary dependence means that the consumption of Western-style diets, typically characterized by an excess of ω-6 PUFAs (15–20 times higher than ω-3), may increase the risk of DHA deficiencies, leading to cognitive dysfunction [5].

Studies have shown that ω-3 PUFAs play a key role in numerous aspects of brain physiology and biological activity, which may explain some of their beneficial effects, including (a) membrane fluidity, because ω-3 PUFAs enhance the fluidity of neuronal membranes due to their “flexible” chemical structure; (b) synaptic function, as ω-3 PUFAs interact directly with membrane-bound proteins, such as enzymes, ion channels and glucose transporters, thereby influencing signal transduction and synaptic activity; (c) inflammation, where ω-3 PUFAs serve as precursors to anti-inflammatory eicosanoids and induce the synthesis of resolvins and neuroprotectins; (d) cerebral blood flow, with established effects of ω-3 PUFAs on endothelial function that may impact cerebral blood flow; and (e) neurotrophic effects, wherein ω-3 PUFAs slow the degradation of neural tissue, have potent anti-apoptotic effects and support the maintenance of healthy axons and synaptic structures [6].

In spite of their crucial role in brain structure and function, the high content of unsaturated fatty acids in brain membranes, combined with the brain’s high oxygen consumption, makes the brain particularly susceptible to lipid peroxidation, with some regions more vulnerable than others, like the cerebral cortex, due to extremely high oxygen consumption, and the cerebellum, specifically, the Purkinje cells [7]. Lipid peroxidation is a primary consequence of free-radical-induced damage that directly harms cell membranes, detrimentally affecting their integrity and function [8]. It also generates several toxic secondary products, including 4-hydroxynonenal (HNE), 4-hydroxyhexenal (HHE), malondialdehyde (MDA) and acrolein, which are highly reactive and can bind to cellular proteins, often negating their function [9]. Consequently, lipid peroxidation can trigger the formation of protein carbonyls, which are irreversible oxidative post-translational modifications (oxPTMs) that usually result in the loss of protein functionality and can eventually lead to the formation of cytotoxic aggregates and, ultimately, cell death [10]. Products generated from the oxidation of reducing sugar are also a source of protein carbonyls [11], which can also be formed by the direct attack from reactive oxygen species (ROS), the metal-catalyzed oxidation of certain amino acids and the direct oxidation of tryptophan [12]. An increasing body of evidence suggests that toxic lipid peroxidation products and protein carbonylation are involved in the pathogenesis of nearly all neurodegenerative diseases, occurring in normal aging [13,14] and other pathologies related to cognitive decline, such as obesity and type 2 diabetes [15,16]. It is worth mentioning that protein carbonylation, in addition to serving as a marker of protein oxidation, plays a significant role in metabolic regulation. A detailed study of this process may contribute to understanding the mechanisms underlying the pathogenesis of numerous diseases characterized by elevated levels of oxidative stress [17].

On the other hand, ω-3 PUFAs have been demonstrated to possess antioxidant properties because they can regulate the heme-oxygenase-1 (HO-1), the nuclear factor erythroid 2 like 2 (NFE2L2), increasing the formation of reduced glutathione (GSH) and antioxidant enzymatic activity [18]. Accordingly, several studies have associated the intake of ω-3 PUFAs with lower levels of oxidative damage in the brain [19,20,21] and the selective protection from oxidation of proteins in the cerebral cortex [22] and the cerebellum [23] in obese rats.

Despite the beneficial effects of ω-3 PUFAs being observed for both EPA and DHA, it is well documented that these two PUFAs are not interchangeable. Previous studies have demonstrated that fish oils with varying proportions of EPA and DHA possess different capacities to modulate lipid metabolism, decrease cardiovascular risk and regulate inflammation and the antioxidant system in metabolic tissues [24]. Likewise, EPA and DHA have shown different effects on neuronal cells [25]. The application of ω-3 PUFAs in nutritional strategies to promote brain health must address the controversy surrounding their role in redox homeostasis and other parameters, as well as the need to determine the optimal consumption of these highly unsaturated fatty acids to maximize their benefits without causing any adverse effects on the CNS.

Therefore, the aim of this study was to evaluate the influence of the degree of unsaturation of the fatty acids of different oils on redox status in the cerebral cortex and the cerebellum of healthy male rats. The research specifically addressed the effects of dietary supplementation with a fish oil containing an increased concentration of DHA (80%) compared to a fish oil containing 25% of DHA and 25% of EPA, a soybean oil containing an increased concentration (50%) of the less unsaturated ω-6 linoleic acid and an oil containing an increased concentration of saturated fat (coconut oil) on oxidative stress and the carbonylated proteome in the cerebral cortex and the cerebellum of healthy male rats.

## 2. Materials and Methods

### 2.1. Animal Model and Experimental Design

Forty male Sprague Dawley rats (Hsd:Sprague Dawley^®^ SD^®^, Inotiv, Inc., Indianapolis, IN, USA), aged 22 weeks and weighing around 400 g, were paired and housed in Makrolon cages (425 mm × 265 mm × 180 mm) under controlled environmental conditions, including a temperature of 22 ± 2 °C, humidity ≈ 60% and a 12 h artificial light/dark cycle.

Following an acclimatization phase, the rats were divided into four groups, each one consisting of 10 rats (*n* = 10), and were subjected to different dietary interventions. The diets included a standard diet (Teklad Global 14% Protein Rodent Maintenance Diet, Inotiv, Inc., Indianapolis, IN, USA) supplemented with coconut oil as a control group, containing 90% of saturated fat, soybean oil, EPA/DHA in a 1:1 ratio (with EPA + DHA representing 50% of the fatty acids in the oil), or fish oil, containing 80% DHA, over a period of 10 weeks. Oil supplementation took place twice a week, and a gastric probe was used, delivering the oils at a dose of 0.8 mL/kg body weight (0.74 mg/kg body weight). Coconut oil and soybean oil were provided by Fauser Vitaquellwerk KG (Hamburg, Germany) and Clearspring Ltd. (London, UK), respectively. The dosage of the DHA supplement in this study was selected according to the recommendation of the European Food Safety Authority for the general European population [26], with dose translation based on body surface area, as detailed in Reagan-Shaw et al. [27].

Throughout the study, rats had ad libitum access to both food and water. Details of the diet composition and the fatty acid profiles of each supplement are shown in Supplementary Tables S1 and S2.

At the end of the experiment, rats were anesthetized using ketamine chlorhydrate and xylacine (80 mg/kg and 10 mg/kg body weight, respectively) and then sacrificed by exsanguination. Blood samples were taken via cardiac puncture. Plasma and erythrocytes samples were obtained through centrifugation of blood, aliquoted and stored at −80 °C until use, as previously described [24]. The cerebral cortex and the cerebellum were washed with a solution of 0.9% NaCl after extraction at 4 °C. Afterward, they were weighed, submerged in liquid nitrogen and stored at −80 °C. All the analyses described in the manuscript were conducted within the first six months of sacrificing the rats.

These rats were the same animals used in the previous study [24]. To facilitate the reading of the current study, the main biochemical and physiological details of the rats are shown in Supplementary Table S3. In short, rats supplemented with the fish oil containing 80% DHA had decreased ectopic fat accumulation and plasma total cholesterol in comparison with rats receiving coconut and soybean oil supplements. Moreover, this fish oil containing 80% DHA enriched the cell membranes in both DHA and EPA while decreasing the arachidonic acid (ARA) content and led to an improvement in the antioxidant defense in metabolic tissues.

### 2.2. Fatty Acid Extraction and Profile Analysis

Lipids from brain tissue were extracted and quantified according to the Bligh and Dyer [28] procedure, and fatty acid profiles were assessed using the method outlined by Lepage and Roy [29], as detailed in previous publications [22,23].

### 2.3. Analysis of Lipid Peroxidation Products

The determination of conjugated dienes hydroperoxides followed the method developed by the American Oil Chemists’ Society (AOCS) [30], while the quantification of oxidized phospholipids (oxPLs), 4-HNE, 4-HHE and MDA, both free and bound to proteins, was conducted through a dot-blot immunoassay, as previously described [22,23].

### 2.4. Analysis of Protein Glutathionylation

Analysis of protein-SSG adducts was accomplished using the same dot-blot immunoassay protocol as the one employed for lipid peroxidation products bound to proteins, as well as the same sample coming from the remaining protein pellets from the Bligh and Dyer extraction, as described above. After spotting and blocking membranes for 1 h, membranes were incubated using anti-GSH (1:1000). After overnight incubation, membranes were incubated with FITC-labeled secondary antibody (1:1000). Afterward, membranes were Ponceau-stained.

### 2.5. Analysis of Total and Specific Protein Carbonylation

Protein carbonylation measures in the cortex and the cerebellum were conducted by following the protocol detailed in [22,23], based on the labeling of protein carbonyl moieties with the fluorescence probe fluorescein-5-thiosemicarbazide (FTSC), isolation of proteins in 1D or 2D electrophoresis gels and identification of carbonylated proteins via nano-LC ESI-IT-MS/MS, using a Dionex UltiMate 3000 Series (ThermoFisher, Rockford, IL, USA) equipment coupled with a dual-pressure linear ion trap mass spectrometer LTQ Velos Pro with electrospray ionization (ESI) (Thermo Fisher, Rockford, IL, USA).

Protein extraction from the brains was performed within six months, followed by protein carbonyl analysis according to the described protocol. The original protein extracts were aliquoted and stored at −80 °C. For carbonyl analysis, an aliquot of each extract was labeled with FTSC, which not only labels but also stabilizes the protein carbonyls. This procedure was performed in triplicate, with one aliquot derivatized at a time to avoid repeated freeze–thaw cycles of the raw protein extract. All carbonyl analyses were completed within one month of derivatization.

### 2.6. Carbonylated Proteome and Functional Enrichment Analysis of Rat Cerebral Cortex and Cerebellum

Functional enrichment analyses of the carbonylated proteome in rats’ brains (specifically the brain cortex and cerebellum) were carried out separately by inputting the relevant gene list of respective carbonylated proteins into the freely available Search Tool for the Retrieval of Interacting Genes (STRING) software version 11.5 (http://stringdb.org/, accessed on 1 September 2024) and selecting *Rattus norvegicus* as the organism. Enrichment in significant gene ontology (GO) terms and KEGG pathways was considered when the false discovery rate (FDR) was less than 0.05, indicating the *p*-value corrected for multiple testing using the Benjamini–Hochberg procedure [31,32].

### 2.7. Antioxidant Status and Enzymatic Activities in Brain

Superoxide dismutase (SOD) and catalase (CAT) activities were measured in total brain according to the methods developed by Mirsa and Fridovich [33] and Cohen et al. [34], respectively. Glutathione peroxidase (GPx) and glutathione reductase (GR) activities in the brain were assessed by following the method developed by Wheeler et al. [35], while GSH and GSSG were measured according to Hissin and Hilf [36]. The GSSG/GSH ratio was also calculated as a biomarker of the redox state. Details of the adapted protocols can be consulted in [24].

### 2.8. Image Analysis and Densitometry Measures

The exposure of dot-blot membranes and gel images was conducted in the UVP BioDoc-It2 Gel Imaging System UV transilluminator (Analytik Jena AG, Upland, CA, USA) equipped with a 520 nm band-pass filter (520DF30 62 mm).

Dot-blot membranes were analyzed using the free software tool ImageJ 1.54k (Wayne Rasband—NIH, Bethesda, MD, USA, freely available for download at https://imagej.net/ij/). LabImage 1D (Kapelan Bio-Imaging Solutions, Halle, Germany) was employed to analyze the 1D gel images. Analysis of 2D gel images was carried out using PDQuest software version 7.4 (Bio-Rad, Hercules, CA, USA).

### 2.9. Statistics

Data in the tables and figures are presented as means with standard deviations (SDs) (*n* = 10). One-way and two-way analyses of variance (ANOVA) were conducted to assess the effects of different oils administered to the rats on the brain, the brain cortex and the cerebellum, as well as to examine differences between the brain cortex and cerebellum. Post hoc analyses were performed to compare group means when a significant effect was identified in the ANOVA using Tukey’s honestly significant difference (HSD) test, with significance set at *p* < 0.05. Significant differences between the means are indicated by superscripts a, b and c. In comparisons of the carbonylated proteome between the cortex and cerebellum, significant differences in the quantity and carbonylation index of common proteins were evaluated using Student’s *t*-test (*n* = 40). Assumptions of normality and homogeneity of variance were verified prior to analysis using the Shapiro–Wilk and Levene tests, respectively. All statistical analyses were performed using Jamovi Version 2.3.18.0 (University of Auckland, Auckland, New Zealand, freely available for download at https://www.jamovi.org).

### 2.10. Materials and Reagents

The fish oil, with EPA/DHA in a 1:1 ratio and 50% EPA + DHA of total fatty acids, was a blend of AFAMPES 121 EPA (210 mg DHA/g fish oil; 55 mg EPA/g fish oil; AFAMSA, Vigo, Spain) and Omega-3 RX (250 mg DHA/g fish oil; 460 mg EPA/g fish oil; EnerZona, Milan, Italy). The fish oil with 80% DHA was purchased from IFIGENEQUIP 98 S.L. (Barcelona, Spain).

Various chemicals and reagents were employed in the study, including DTT, PMSF, EDTA, iodoacetamide, Tris–HCl, N,N,N′,N′-Tetramethyl ethylenediamine (TEMED), TCA, CHAPS and BCA assay from Sigma (St. Louis, MO, USA). Additional supplies, such as BlueBlock Blocking Solution, PBS 10× solution, PBS with 0.05% Tween^TM^-20 (PBST) 10× solution and Protease inhibitor ProteoBlock, were obtained from Serva (SERVA Electrophoresis GmbH, Germany) and Thermo Fisher Scientific Inc. (Rockford, IL, USA), respectively. Ketamine-HCl and xylazine (Rompun 2%) anesthetics were acquired from Merial Laboratorios S.A. (Barcelona, Spain) and Química Farmacéutica S.A. (Barcelona, Spain), respectively. FTSC was procured from Invitrogen (Carlsbad, CA, USA), and sequencing-grade trypsin for protein digestion came from Promega (Madison, WI, USA).

The E06 mouse monoclonal antibody (IgM), labeled with TopFluor^®^ for oxPL, was provided by Avanti^®^ Polar Lipids, Inc. (Croda International Plc, Birmingham, AL, USA). Primary antibodies, including anti-MDA (goat polyclonal antibody to malondialdehyde), anti-4-HNE (mouse monoclonal (HNEJ-2) antibody to 4-HNE), anti-4-HHE (mouse monoclonal antibody to 4-HHE) and anti-GSH (mouse monoclonal antibody to GSH), along with secondary antibodies, such as donkey anti-goat IgG (H + L) (FITC) and goat anti-mouse IgG (H + L) (FITC), were purchased from Antibodies.com (Europe AB, Stockholm, Sweden). Bio-Rad Laboratories (Hercules, CA, USA) supplied the Bio-Rad protein assay, acrylamide, bis-N,N-methylene-bis-acrylamide and PVDF membranes. For 2D electrophoresis, IPG strips (Immobiline DryStrip gels) with pH ranges of 3–10 and lengths of 7 and 18 cm were obtained from GE Healthcare Bio-Sciences AB (Uppsala, Sweden), along with IPG buffer 3–10 pH and pharmalyte 3–10 pH. Bromophenol blue PlusOne was provided by Cytiva (Marlborough, MA, USA). Ponceau-S-Solution was purchased from Sigma-Aldrich^®^ (Merck KGaA, Darmstadt, Germany).

## 3. Results and Discussion

### 3.1. Comparison of the Lipid Profile Between Cortex and Cerebellum and Response to the Degree of Unsaturation of Fatty Acids in Oils Administered to the Rats

The lipid composition of the brain plays a crucial role in its metabolism and overall functioning. As shown in Table 1, the analysis of the lipid composition of the brains of 8-month-old male Sprague Dawley rats revealed that the cerebellum is a slightly, yet significantly, fattier region than the cerebral cortex. This may be explained by the different relative distributions of gray and white matter in these areas, with the cortex exhibiting the lowest lipid levels due to its higher proportion of gray matter, as previously reported [37].

Significant differences in the fatty acid classes between both regions were also evident, with a higher amount of saturated fatty acids (SFAs) and PUFAs in the cortex, while the cerebellum accumulated more monounsaturated fatty acids (MUFAs). Interestingly, the cerebellum presents a slightly higher proportion of ω-3 PUFAs, particularly DHA, and a marked decrease in ω-6 fatty acids, primarily due to the lower amount of ARA in this region. The lipid composition in rats from this study was similar to that previously described for 8-month-old Sprague Dawley rats by Firląg et al. [38], although these authors reported a higher proportion of DHA and lower MUFA levels in both the cortex and the cerebellum. The different basal diet used by these authors may explain this variation.

Regarding the effects of the oils, the results demonstrated that the degree of fatty acid unsaturation did not change the proportion of SFAs, MUFAs and PUFAs but significantly altered the fatty acid profiles within each category, especially the profiles of PUFAs. As shown in Table 1, both fish oils, particularly the one containing 80% DHA, increased DHA and decreased ARA levels in both the cortex and the cerebellum, which responded similarly to supplementation in terms of fatty acid profiles. Similar enrichments of ω-3 PUFAs in the cortex and cerebellum due to fish oil supplementation have been previously described in rats fed standard and obesogenic diets [22,23,38], as well as in cows [39] and in mice with Alzheimer’s disease [40]. In these studies, such enrichments have been directly associated with improvements in cognitive performance in obesity, aging and neurodegenerative diseases.

### 3.2. Redox Status in the Cortex and the Cerebellum and Response to the Degree of Unsaturation of Fatty Acids in Oils Administered to the Rats

The CNS is highly susceptible to oxidative stress for several reasons, including its high oxygen consumption (more than 20% of the total body oxygen budget), the abundance of mitochondria, the significant reliance on free radical signaling for proper brain function, which necessitates precise regulation of redox homeostasis, the high degree of unsaturation of fatty acids and relatively modest antioxidant defenses [7]. This modest antioxidant defense in CNS tissue, as described in the literature, was confirmed in the current study. Accordingly, the activities of the antioxidant enzymes SOD, CAT, GPx and GR in the brain (Table 2) were significantly lower than those in the liver of the same cohort of rats [24]. The brain also contained lower amounts of glutathione, although the GSSG/GSH ratio was quite similar in both tissues. Furthermore, the antioxidant defense in the brain was lower than that measured in perigonadal adipose tissue [24]. This may be a consequence of the crucial role of free radicals and the need for optimal production in sufficient amounts to maintain normal brain function, as mentioned earlier, particularly neurotransmission, which may justify why the brain has constitutively lower levels of antioxidant activity.

On the other hand, supplementation with the two fish oils, especially with the one containing 80% DHA, increased the GSH amount, which is the main antioxidant in the brain [41], presenting lower GSSG/GSH ratio and boosting the coordination of the SOD, GPx, and CAT enzyme activities for the most efficient elimination of ROS. This is evidenced by the lowest SOD/GPx + CAT ratio in the group supplemented with 80% DHA.

Regarding the formation of oxidized products of lipids and proteins shown in Table 3, the results clearly indicated that the cerebral cortex contained amounts of oxidized lipids and proteins at steady-state levels in healthy conditions. Therefore, the cortex showed significantly higher levels of secondary lipid peroxidation products, such as MDA, HNE, HHE and also oxPC, both free and adducted to proteins, as well as an increased grade of protein carbonylation than the cerebellum. Accordingly, the cerebral cortex also presented higher levels of protein glutathionylation, which is a reversible oxidative modification of the protein thiols (Pr-SH) that occurs during oxidative stress, and so, these data agreed with the results of oxidized lipid and protein formation in both brain regions. However, it should be noted that the Pr-SSG can be reduced back to protein thiols by Grx1 or Trx1. The GSSG formed in this reaction is effectively reduced to GSH by GR, being considered a reservoir of GSH while protecting proteins from irreversible oxidative modification [42]. Concerning the effects of the oils, the results demonstrated that the increasing degree of unsaturation of the oils did not increase lipid and protein oxidation either in the cortex or in the cerebellum. In fact, supplementation with the most unsaturated ω-3 fish oil, namely 80% DHA, tended to decrease protein carbonylation, especially in the cortex, in agreement with the effects of this oil in promoting antioxidant defense, as said before.

The cerebellum of healthy rats exhibited lower levels of oxidized lipid and protein compared to the cerebral cortex at steady-state levels, despite cerebellum containing more fat and fat that was more unsaturated (higher proportion of ω-3 PUFAs) (Table 1). The highly dynamic and region-specific oxygen consumption in the brain may explain these results. The cerebral cortex contains a higher proportion of gray matter than the cerebellum, and gray matter consumes over twice the amount of oxygen compared to white matter, with the highest consumption observed in the medial occipital lobe [43].

Despite the lower basal levels of oxidative stress found in the cerebellum compared to the cortex, several studies have indicated that the cerebellum is one of the most susceptible regions of the brain to oxidative insults [44]. This may be due to the lower levels of GSH in cerebellar astrocytes compared to cortical astrocytes [45]. Although the GSH data presented in this study (Table 2) pertain to the whole brain, our data on protein glutathionylation (Table 3) support this finding, as this modification is significantly more abundant in the cortex than in the cerebellum, likely due to its higher production in this region.

The increased vulnerability of the cerebellum to oxidative insults has been previously noted. For instance, cerebellar neurons were shown to be more sensitive to ROS induced by methylmercury toxicity [45] compared to cortical neurons. Additionally, the cerebellum demonstrated increased sensitivity to oxidative stress induced by the consumption of obesogenic diets, exhibiting higher levels of lipid and protein oxidation [22,23]. Based on the results of the current study, it can be inferred that the increased vulnerability of the cerebellum is largely independent of the high proportion of ω-3 in this region and may be attributed to its modest antioxidant defenses. This conclusion arises from two observations: first, the cerebellum has low basal levels of oxidation despite its higher unsaturated fat content, and second, the enrichment in ω-3 achieved through supplementation with EPA/DHA in a 1:1 ratio, and even the higher unsaturation degree of 80% DHA fish oil, did not elevate oxidative damage; rather, it seemed to exacerbate the poor antioxidant defenses of the cerebellum. Therefore, both fish oils, particularly the 80% DHA, were harmless to the brain as well as to the rest of the body, as previously demonstrated [24]. The excess unsaturation appears to be offset by the stimulation and improved synchronization of the endogenous antioxidant enzymatic system while still preserving the beneficial properties of ω-3 in the brain and throughout the body, as supported by the phenotypic health data measured in these rats.

Another important outcome of incorporating higher amounts of ω-3 into brain membranes is the potential enhancement of the brain’s resilience to potential oxidative insults caused by natural aging, exogenous factors (such as pollution, toxins, pathogens, etc.) or the consumption of obesogenic diets, as previously suggested [22,23].

### 3.3. Comparison Between the Carbonylated Proteome of the Cortex and Cerebellum and Response to the Degree of Unsaturation of Fatty Acids in Oils Administered to the Rats

#### 3.3.1. Comparison Between the Cortical and Cerebellar Proteome and Carbonylated Proteome

The carbonylated proteome in the cortex and cerebellum of healthy rats at steady-state levels was identified and quantified in the present study. A total of fifty-six proteins in the cortex and fifty-seven proteins in the cerebellum were determined to be carbonylated under these conditions. The list of the carbonylated proteins identified is shown in Supplementary Table S4 and Supplementary Figures S1–S4. These carbonylated proteins correspond to the same proteins identified in the cerebral cortex [22] and cerebellum [23] of rats subjected to high-fat and high-sucrose diets, thereby confirming the stability of the carbonylated proteome in terms of its protein composition, although the extent of carbonylation for each protein is highly contingent upon the redox environment.

Consequently, in alignment with previous data obtained from obese rats, our results corroborate that the carbonylated proteome in the cortex is enriched in proteins involved in various KEGG pathways, including energy production pathways (such as glycolysis/gluconeogenesis, pyruvate metabolism and the TCA cycle), amino acid metabolism pathways (such as cysteine, methionine, arginine, alanine, aspartate and glutamate metabolism, along with arginine biosynthesis) and multiple signaling pathways, including, but not limited to, the hypoxia-inducible factor (HIF)-1 signaling pathway, hippo signaling pathway, PI3K-Akt signaling pathway and glucagon signaling pathways. Additionally, gap and tight junctions, the phagosome, the cell cycle, various forms of cell death and proteins directly associated with neurodegenerative diseases were also identified as enriched in carbonylated proteins and potentially regulated by this oxidative post-translational protein modification (oxPTMs) within the rat cortex. Regarding the carbonylated proteome in the rat cerebellum, similar pathways enriched in carbonylated proteins were observed. Notably, significant enrichments were found in proteins associated with energy production (including glycolysis/gluconeogenesis, pyruvate metabolism and the TCA cycle), amino acid metabolism (involving arginine, proline, cysteine, methionine, alanine, aspartate and glutamate metabolism), as well as the HIF-1 and glucagon signaling pathways, adherens and tight junctions, focal adhesion and proteins implicated in neurodegenerative diseases.

Despite the similarities in the overarching pathways potentially regulated by carbonylation, an in-depth examination of the specific proteins identified in each carbonylated proteome revealed only partial overlap. Furthermore, the current study’s findings indicating elevated oxidative stress in the cortex compared to the cerebellum at steady-state levels invite a more cautious comparison of both carbonylated proteomes. Therefore, beyond delineating the carbonylated proteome in both brain regions, this study undertakes both qualitative and quantitative comparisons of the cortical and cerebellar carbonylated proteomes, encompassing relative protein abundance and carbonylation status.

**Figure 1:**
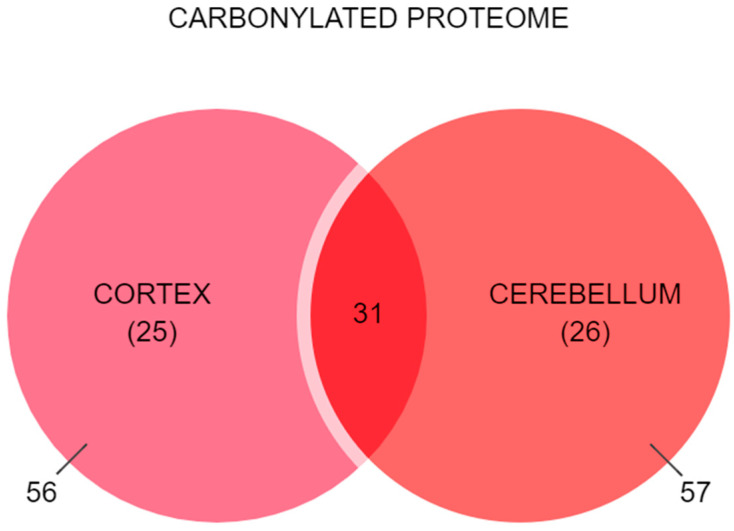
Comparison between the carbonylated proteomes identified in the cortex and the cerebellum of 8-month-old Sprague Dawley rats. Created using https://www.statskingdom.com/venn-diagram-maker.html (accessed on 1 September 2024).

The qualitative comparison of the carbonylated proteomes in each brain region (Supplementary Table S4) indicated that almost 30% of the total carbonylated proteins identified (31 proteins out of 113) were present in both the cortex and the cerebellum. The remaining proteins were exclusively identified and quantified in one of each region, i.e., 25 proteins in the cortex and 26 proteins in the cerebellum (Figure 1).

The proteins which were found carbonylated in both the cortex and the cerebellum and successfully quantified in terms of protein amount and protein carbonylation index are shown in Table 4.

In view of the data shown in Table 4, one of the most significant differences between the cortical and cerebellar carbonylated proteome was found in proteins directly related to ATP production. The creatine kinase B-type (Ckb) protein catalyzes the reversible transfer of phosphate between ATP and various phosphagens (e.g., creatine phosphate), playing a crucial role in energy transduction in tissues with large and fluctuating energy demands, such as the brain [46]. In this study, Ckb was almost twice more abundant in the cortex than in the cerebellum, being also significantly more oxidized and one of the main components of the carbonylome in this region. Otherwise, the cerebellum showed a higher relative level of the mitochondrial ATP synthase subunit beta—higher than its Ckb content and higher than the level found in the cortex; therefore, the production of ATP through this means seems to be more important in the cerebellum. Moreover, the ATP synthase was significantly more oxidized in the cerebellum than in the cortex, being one of the main targets of oxidation in this region. Accordingly, the cerebellum also contained higher relative amounts of the glycolic proteins directly involved in pyruvate metabolism, ATP production and the TCA cycle, such as pyruvate kinase, (Pkm), pyruvate dehydrogenase E1 component subunit alpha (Pdha1) and beta (Pdhb), mitochondrial aconitate hydratase (Aco2), cytoplasmic aspartate aminotransferase cytoplasmic (Got1) and L-lactate dehydrogenase B chain (Ldha), an enzyme participating in anaerobic glycolysis [47]. The generally higher oxidation of these enzymes agreed with the idea that the cerebellum may be more dependent on this process for ATP production than the cortex in physiological conditions. Supporting this idea, the carbonylated proteins found exclusively in the cerebellum were mainly involved in the ATP production (Supplementary Table S4).

Several differences were found in other glycolic enzymes. Although some of these enzymes remained unchanged (Aldoc and Gapdh) in both expression and carbonylation ratios, the cerebellum contained higher amounts of alpha-enolase but less fructose-bisphosphate aldolase A (Aldoa) than the cortex, although no differences in carbonylation were detected. Interestingly, no differences in expression or carbonylation in another member of the enolase family, the gamma-enolase, were detected between the cortex and the cerebellum at steady-state levels. This stability of gamma-enolase may be explained by its critical neurotrophic and neuroprotective properties in a broad spectrum of CNS neurons [48]. On the other hand, the oxidation of triosephosphate isomerase (Tpi1) was higher in the cortex, perhaps as a direct consequence of its superior oxygen consumption.

Other groups of proteins found carbonylated both in the cortex and the cerebellum were related to glutamate, a major excitatory neurotransmitter of the vertebrate central nervous system [49]. These proteins were cytoplasmic Got1, glutamine synthetase (Glul) and mitochondrial glutamate dehydrogenase 1 (Glud1). The three proteins can act as scavengers of neurotoxic glutamate in brain neuroprotection [50], and the data shown in Table 4 indicate that these enzymes are more abundant in the cerebellum. Accordingly, glutamate serves as the primary neurotransmitter for some localized brain regions, such as cerebellum granule cells [51].

Significant differences were also found in proteins directly related to neurotransmission dynamics and neuronal growth. A higher proportion of proteins directly involved in neurotransmission were found in the cerebral cortex as compared to the cerebellum, in agreement with the exceptionally high neural activity derived from its role in controlling the higher cognitive functions [52]. Indeed, the cerebral cortex has relatively higher amounts of tubulin beta-2A chain (Tubb-2A), representing an important part of the total carbonylated proteome. Tubulins are major constituents of cytoskeletal microtubules. Our data revealed that tubulins are also one of the main targets of carbonylation in the cortex as compared to the cerebellum. Several other isoforms of tubulins were found exclusively carbonylated in the cortex, as shown in Supplementary Table S4, and their relative abundances were also higher than in the cerebellum. On the contrary, the cerebellum had a higher cytoplasmic actin content than the cortex, with a higher ratio of carbonylation. The carbonylation rates of septin-11 were also slightly superior in the cerebellum, and several other isoforms of septins were also identified as part of the cerebellar carbonylated proteome (Supplementary Table S4). Septins have been associated with GABAergic synaptic connectivity, being the GABAergic neurons located in the nuclei of the cerebellum [53]. In addition to cytoskeletal proteins, the cerebral cortex also contained higher amounts of other proteins critical in neurotransmission activity, including dihydropyrimidinase-related protein 2 (Dpysl2), guanine nucleotide-binding protein G(o) subunit alpha (Gnao1), synapsin-1 (Syn1) and syntaxin-binding protein 1 (Stxbp1), although they were not more oxidized than in the cerebellum.

Finally, among the shared carbonylated proteins between the cortex and the cerebellum, there were higher amounts but same oxidations of some isoforms of chaperons, the heat shock cognate 71 kDa (Hspa8) and 60 kDa heat shock protein mitochondrial (Hspd1), while the cerebral cortex contained more ubiquitin carboxyl-terminal hydrolase isozyme L1 (Uchl1), a protein related to the ubiquitin system within the protein turnover process [54].

Therefore, the carbonylated proteome in the cortex is mainly constituted by proteins related to the rapid ATP production and neurotransmission dynamics, while there is a high proportion of proteins from glycolysis (both aerobic and anaerobic) and the TCA cycle in the cerebellum.

#### 3.3.2. Influence of the Degree of Unsaturation of Fatty Acids in Oils Administered to the Rats on the Carbonylated Proteome

The isolation of carbonylated proteins from the cortex and cerebellum, along with the individual quantification of their carbonylation levels, revealed significant changes in the carbonylation rate of certain proteins due to supplementation with oils at increasing levels of unsaturation. This modulation was detected in both the cortex and cerebellum, although there were no changes in the overall carbonylation levels of the proteome, except for a statistically significant trend in cortical proteins exhibiting a decrease in carbonylation levels in the group supplemented with 80% DHA compared to the soybean-oil-supplemented group (Table 3). No differences in the concentration of any protein were measured due to different supplementation.

**Figure 2:**
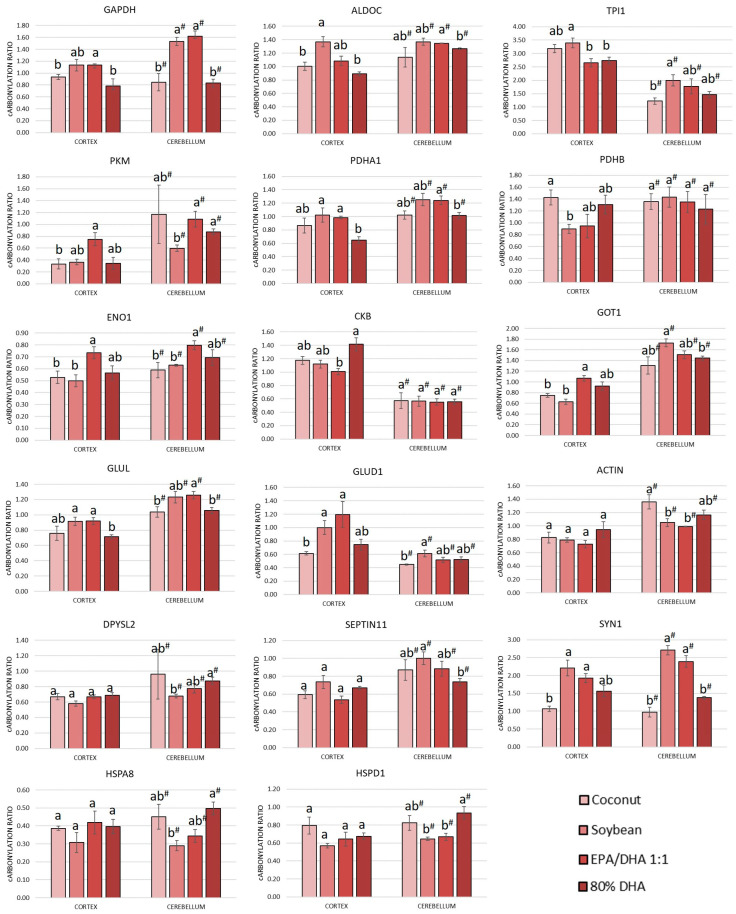
Carbonylated proteins modulated by supplementation of a diet with oils at increasing degree of fatty acid unsaturation and found carbonylated in both the cortex and the cerebellum of 8-month-old Sprague Dawley rats. Bars represent means ± standard deviations (*n* = 10). One-way ANOVA analyses were conducted. Post hoc Tukey HSD analyses were performed following ANOVA to assess mean differences between groups. Means with the same superscript letter (a, b in the cortex and a^#^, b^#^ in the cerebellum) are not significantly different from each other, while means with different letters indicate significant differences, according to Tukey’s post hoc test (*p* < 0.05). Protein abbreviations are outlined in Table 4.

**Figure 3:**
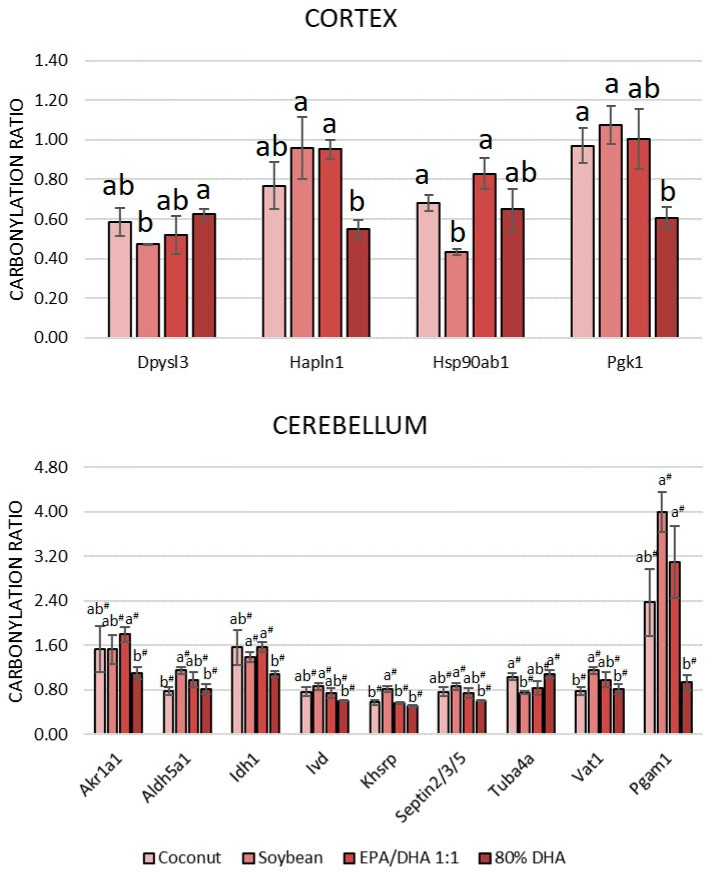
Carbonylated proteins modulated by supplementation of a diet with oils at increasing degree of fatty acid unsaturation in the cortex (**upper panel**) and the cerebellum (**bottom panel**) of 8-month-old Sprague Dawley rats. Bars represent means ± standard deviations (*n* = 10). One-way ANOVA analyses were conducted. Post hoc Tukey HSD analyses were performed following ANOVA to assess mean differences between groups. Means with the same superscript letter (a, b in the cortex and a^#^, b^#^ in the cerebellum) are not significantly different from each other, while means with different letters indicate significant differences, according to Tukey’s post hoc test (*p* < 0.05). Dpysl3, Dihydropyrimidinase-Related Protein 3; Hapln1, Hyaluronan and Proteoglycan Link Protein 1; Hsp90ab1, Heat Shock Protein HSP 90-beta; Pgk1, Phosphoglycerate Kinase 1; Akr1a1, Aldo-Keto Reductase Family 1 Member A1; Aldh5a1, Succinate-Semialdehyde Dehydrogenase Mitochondrial; Idh1, Isocitrate Dehydrogenase [NADP] Cytoplasmic; Ivd, Isovaleryl-CoA Dehydrogenase Mitochondrial; Khsrp, Far Upstream Element-Binding Protein 2; Septin2/3/5, Septin-2/-5 and Neuronal-Specific Septin-3; Tuba4a, Tubulin Alpha-4A Chain; Vat1, Synaptic Vesicle Membrane Protein VAT-1 Homolog; Pgam1, Phosphoglycerate Mutase 1.

The results indicated that nearly 29% of the carbonylated proteome from the cortex (16 out of 56 carbonylated proteins) and more than 42% of the carbonylated proteome from the cerebellum (24 out of 57 carbonylated proteins) exhibited alterations in their carbonylation levels due to the different oils. A high percentage of proteins that responded in some manner to the supplementation were proteins that were found to be carbonylated in both the cortex and the cerebellum (57%, 17 out of the 30 carbonylated proteins that responded to supplementation). The carbonylation ratios of these proteins in each region and dietary group are shown in Figure 2. Additionally, proteins that responded to supplementation and were exclusively found carbonylated in the cortex or in the cerebellum are shown in Figure 3.

As shown in Figure 2, nearly half of the responsive proteins in both the cortex and cerebellum were glycolytic enzymes. These proteins included Gapdh, Aldoc, Tpi1, Pkm, Pdha1, Pdhb and Eno1. In both regions, the level of carbonylation for these proteins generally decreased in the following order: soybean > EPA/DHA 1:1 > coconut > 80% DHA. It should be noted that the higher relative content of EPA in the fish oil containing EPA/DHA 1:1 may explain the observed differences in the modulation of the oxidative state of specific brain proteins compared to the oil containing a higher proportion of DHA. Upon entering the brain, DHA is incorporated into phospholipid membranes [55], whereas EPA undergoes β-oxidation, a process that may lead to increased oxidative stress [56]. A similar case can be observed with soybean oil, which provides elevated amounts of linolenic acid, a fatty acid that is also metabolized through β-oxidation in the brain rather than incorporated to the membrane phospholipids. Regarding the coconut oil, its extraordinarily high content of saturated fat (more than 90%, Supplementary Table S2) makes the oil less susceptible to lipid peroxidation, and in healthy conditions, this can lead to less oxidative stress. In this regard, some authors claim that there exists a misconception that dietary SFAs can cause inflammation, which is related to oxidative stress, and dietary saturated fats seem to be less harmful than unsaturated fats in certain metabolic parameters [57].

Additionally, the glycolytic enzyme phosphoglycerate kinase 1 (Pgk1) in the cortex and the glycolytic enzyme phosphoglycerate mutase 1 (Pgam1) in the cerebellum exhibited a significant reduction in carbonylation in the 80% DHA group compared to the other groups (Figure 3), reinforcing the antioxidant protective effects of DHA in controlling excessive carbonylation of glycolytic enzymes in the brain. Furthermore, and relating to energy production, a reduced level of carbonylation of Ckb was observed in the cortex of rats that received the fish oil supplement with an EPA/DHA ratio of 1:1. As previously mentioned, Ckb is a protein involved in the rapid provision of ATP with prominent importance for the cortex, according to its elevated concentration (Table 4). In this instance, the higher relative proportion of EPA in the supplement appears to be more efficient than that of DHA, linoleic acid or SFAs. This oil has already been demonstrated to reduce the carbonylation levels of this protein in both the cortex [22] and the cerebellum [23] of obese rats fed high-saturated-fat and -sugar diets.

Nearly half of the responsive proteins in both the cortex and the cerebellum were related to neurotransmission (Figure 2). The responsive proteins included proteins involved in amino acid metabolism, synapsis and cytoskeleton dynamics. In general, the level of carbonylation of these enzymes followed the same tendency as that described for glycolytic enzymes, with the soybean and EPA/DHA 1:1 supplemented groups showing more oxidation than groups supplemented with coconut and 80% DHA. The capacity of the fish oil supplement to modulate the carbonylation of proteins involved in neurotransmission and to counteract the effects of the intake of diets high in fat and sugar on the cortex and the cerebellum was previously demonstrated [22,23].

Among the proteins related to amino acid metabolism, there were Got1, Glul and Glud1, which are particularly involved in the metabolism of glutamate, the main excitatory neurotransmitter. Additionally, supplementation with 80% DHA caused significantly less carbonylation in the cytoplasmic enzyme isocitrate dehydrogenase [NADP] (Idh1), belonging to the TCA cycle, and thus, amino acid metabolism, and in the mitochondrial enzyme isovaleryl-CoA dehydrogenase (Ivd), belonging to the leucine catabolic pathway, in the cerebellum (Figure 3).

The proteins related to synapsis and cytoskeleton dynamics that responded to oil supplementation were actin, Dpysl2, Septin 11 and Syn1 (Figure 2), in both the cortex and the cerebellum. Additionally, some other proteins belonging to this category (Figure 3) were found to be sensitive to supplementation, including dihydropyrimidinase-related protein 3 (Dpysl3) and hyaluronan and proteoglycan link protein 1 (Hapln1) in the cortex, and proteins septin-2, -3 and -5, cytoskeletal protein tubulin alpha-4A chain (Tuba4a), synaptic vesicle membrane protein VAT-1 homolog (Vat1) and mitochondrial succinate-semialdehyde dehydrogenase (Aldh5a1). Aldh5a1 catalyzes one step in the degradation of the inhibitory neurotransmitter gamma-aminobutyric acid (GABA) [58] in the cerebellum. It is noteworthy that this group of proteins was found to be more sensitive to supplementation with various oils in the cerebellum than in the cortex. Moreover, while the majority of proteins followed the oxidation trend previously described for the other proteins, showing reduced carbonylation in the group supplemented with a higher percentage of DHA, proteins such as actin, Tuba4a and proteins Dypsl2 and Dypsl3, which are directly implicated in the remodeling of the cytoskeleton [59,60], exhibited an opposite behavior, demonstrating lower levels of carbonylation following supplementation with soybean oil, and to a lesser extent, with EPA/DHA at a 1:1 ratio. It should be noted that actins and other cytoskeletal proteins can function as natural free radical scavengers and can typically withstand certain moderate oxidative damage without losing their molecular function [61]. A similar trend was observed for chaperones Hspa8 and Hspd1 in the cerebellum (Figure 2) and Hsp90ab1 in the cortex (Figure 3).

Finally, supplementation with 80% of DHA caused a significant decrease in the carbonylation of the aldo-keto reductase family 1 member A1 (Akr1a1) protein in the cerebellum (Figure 3). This protein is especially relevant because it functions as a detoxifying enzyme by reducing a range of toxic aldehydes, including lipid-derived aldehydes like acrolein [62]. Its lower level of carbonylation in the 80% DHA supplemented group could facilitate the elimination of these toxic aldehydes to compensate for high unsaturation in the oil. Also in the cerebellum, the far upstream element-binding protein 2 (Khsrp), which was sensitive to different supplementations (Figure 3), followed the same trend as that described for most proteins, with higher levels of carbonylation in the soybean-oil-supplemented group. Although the role of this protein in the cerebellum is not clear yet, Khsrp binds to the dendritic targeting element and may play a role in mRNA trafficking [63].

## 4. Conclusions

This study demonstrated that the degree of unsaturation of fatty acids in oils administered to rats modulates the lipid profiles, antioxidant system and carbonylated proteome of the cerebral cortex and the cerebellum of healthy rats. Dietary supplementation with fish oils, particularly those with a higher percentage of DHA, significantly increased the ω3/ω6 ratio in both the cortex and cerebellum while enhancing antioxidant defenses by elevating the GSH amount. The results showed that an increase in the degree of unsaturation and ω-3 content in the brain did not lead to elevated oxidative stress or the formation of lipid and protein oxidation products. Instead, a trend toward reduced protein damage was observed, particularly with increased DHA supplementation. This effect was more pronounced after analyzing and identifying the specific proteins responsive to dietary supplementation. The majority of sensitive proteins (mainly glycolytic enzymes and proteins involved in neurotransmission) in both the cortex and cerebellum followed this carbonylation trend (in decreasing order): soybean > EPA/DHA 1:1 > coconut > 80% DHA.

Furthermore, the results of this study indicate that, under our experimental conditions, the cerebral cortex exhibits higher levels of oxidative stress compared to the cerebellum. Specifically, the cortex shows significantly greater amounts of lipid peroxidation products, both free and protein-bound, as well as higher levels of protein carbonylation across all experimental groups. These effects were observed regardless of the degree of fatty acid unsaturation in the oils administered to rats. Additionally, our data suggest that the cerebellum has a more limited antioxidant system than the cortex in this rat model, as indicated by the significantly lower level of protein gluthationylation measured in the cerebellum of all rats, irrespective of the type of oil intake. This fact may make the cerebellum more responsive to changes in the cellular redox environment, potentially explaining why a greater number of cerebellar proteins exhibited changes in their carbonylation ratios in response to the degree of unsaturation of fatty acids in the administered oils compared to those observed in the cortex.

Therefore, dietary supplementation with fish oils, particularly those high in DHA, appears to be especially beneficial for enhancing the resilience of both the cortex and cerebellum against potential oxidative insults and natural processes such as aging, which are accompanied by increased cerebral oxidative stress.

## Figures and Tables

**Table 1:** Influence of the degree of unsaturation of fatty acids in oils administered to the rats on the fat and fatty acid composition of the rat cortex and cerebellum ^1^.

	Region	Total ^3^	Coconut	Soybean	EPA/DHA 1:1	80% DHA
Brain weight (g)	-	1.93 (0.05)	2.0 (0.1)	1.9 (0.1)	1.9 (0.1)	1.9 (0.1)
Brain-to-body weight index (%) ^2^	-	0.45 (0.01)	0.46 (0.02)	0.43 (0.02)	0.45 (0.03)	0.44 (0.02)
FAT (%) *	Cortex	6.60 (0.19)	6.69 (0.31)	6.35 (0.13)	6.56 (0.43)	6.80 (0.02)
	Cerebellum	7.64 * (0.18)	7.61 (0.32)	7.82 (0.08)	7.72 (0.27)	7.40 (0.42)
Total SFAs (%) *	Cortex	47.29 (0.37)	47.24 (0.27)	47.37 (0.64)	47.42 (0.32)	47.14 (0.30)
	Cerebellum	43.41 * (0.27)	43.57 (0.32)	43.30 (0.07)	43.40 (0.13)	43.38 (0.46)
Total MUFAs (%) *	Cortex	23.38 (0.73)	23.60 (0.79)	23.16 (1.12)	23.22 (0.75)	23.53 (0.55)
	Cerebellum	30.92 * (0.80)	30.73 (1.16)	30.87 (0.10)	31.18 (0.62)	30.89 (1.28)
Total PUFAs (%) *	Cortex	29.12 (0.20)	28.90 (0.54)	29.39 (0.75)	29.09 (1.06)	29.09 (0.35)
	Cerebellum	25.27 * (0.17)	25.30 (0.93)	25.43 (0.07)	25.02 (0.54)	25.31 (0.88)
ω-6 (%) *^$^	Cortex	15.28 (0.91)	15.87 ^a^ (0.46)	16.21 ^a^ (0.43)	14.72 ^ab^ (0.57)	14.30 ^b^ (0.53)
	Cerebellum	10.88 * (0.77)	11.46 ^c^ (0.46)	11.61 ^c^ (0.16)	10.35 ^cd^ (0.28)	10.09 ^d^ (0.16)
ω-3 (%) *^$^	Cortex	13.84 (0.87)	13.02 ^a^ (0.23)	13.18 ^a^ (0.36)	14.37 ^ab^ (0.50)	14.79 ^b^ (0.38)
	Cerebellum	14.39 * (0.68)	13.84 ^ab^ (0.48)	13.82 ^ab^ (0.09)	14.67 ^b^ (0.62)	15.21 ^b^ (0.94)
ω-3/ω-6 *^$^	Cortex	0.91 (0.12)	0.82 ^a^ (0.03)	0.81 ^a^ (0.01)	0.98 ^ab^ (0.01)	1.04 ^b^ (0.06)
	Cerebellum	1.33 * (0.16)	1.21 ^c^ (0.01)	1.19 ^bc^ (0.02)	1.42 ^d^ (0.09)	1.51 ^d^ (0.11)
ARA (%) *^$^	Cortex	10.16 (0.50)	10.46 ^a^ (0.39)	10.65 ^a^ (0.34)	9.97 ^ab^ (0.44)	9.55 ^b^ (0.42)
	Cerebellum	6.69 * (0.48)	7.04 ^c^ (0.33)	7.15 ^cd^ (0.09)	6.41 ^cd^ (0.18)	6.16 ^d^ (0.15)
DHA (%) *^$^	Cortex	13.60 (0.81)	12.84 ^a^ (0.23)	12.98 ^a^ (0.37)	14.11 ^ab^ (0.53)	14.47 ^b^ (0.37)
	Cerebellum	14.06 * (0.57)	13.60 ^ab^ (0.50)	13.60 ^ab^ (0.10)	14.26 ^ab^ (0.61)	14.78 ^b^ (0.90)

^1^ Values are expressed as mean (standard deviation) (*n* = 10). Two-way ANOVA analyses were conducted. * *p* < 0.05 significant differences due to the factor “tissue” (Cortex and Cerebellum); ^$^ *p* < 0.05 significant differences due to the factor “oil” (Coconut, Soybean, EPA/DHA 1:1 and 80% DHA). Post hoc Tukey HSD analyses were performed following ANOVA to assess mean differences between groups. Means with the same superscript letter (a, b, c, d) are not significantly different from each other, while means with different letters indicate significant differences, according to Tukey’s post hoc test (*p* < 0.05). ^2^ The brain-to-body weight index is brain weight [g]/body weight [g] × 100. ^3^ “Total” refers to the mean (SD) obtained from the two-way ANOVA analysis for each measurement across all individuals in the respective brain region, regardless of the oil received.

**Table 2:** Influence of the degree of unsaturation of fatty acids in oils administered to the rats on antioxidant enzymatic activities and glutathione in brain ^1^.

	Total ^2^	Coconut	Soybean	EPA/DHA 1:1	80% DHA
Brain					
SOD (U/g brain)	663.75 (43.78)	671 (127)	702 (190)	601 (111)	681 (118)
CAT (mmol H_2_O_2_ decomposed/g brain)	0.060 (0.01)	0.059 (0.014)	0.052 (0.016)	0.049 (0.027)	0.061 (0.024)
GPx (U/g brain)	0.70 (0.05)	0.71 (0.09)	0.68 (0.13)	0.65 (0.12)	0.76 (0.07)
SOD/CAT	11,062.50	11,372.88	13,500.00	12,265.31	11,163.93
SOD/GPx	948.21	945.07	1032.35	924.62	896.05
SOD/GPx + CAT	873.36	872.56	959.02	859.80	829.48
GR (U/g brain) ^$^	0.78 (0.15)	0.66 ^a^ (0.14)	0.96 ^b^ (0.38)	0.66 ^a^ (0.17)	0.84 ^ab^ (0.06)
GSH (µmol/g brain) ^$^	0.28 (0.03)	0.26 ^ab^ (0.03)	0.24 ^a^ (0.04)	0.30 ^bc^ (0.04)	0.30 ^c^ (0.04)
GSSG (µmol/g brain)	0.11 (0.01)	0.11 (0.03)	0.12 (0.03)	0.11 (0.02)	0.09 (0.01)
GSSG/GSH ratio ^$^	0.41 (0.08)	0.44 ^ab^ (0.12)	0.50 ^a^ (0.15)	0.36 ^bc^ (0.08)	0.32 ^c^ (0.08)

^1^ Values are expressed as mean (standard deviation) (*n* = 10). One-way ANOVA analyses were conducted. ^$^ *p* < 0.05 significant differences due to the factor “oil” (Coconut, Soybean, EPA/DHA 1:1 and 80% DHA). Post hoc Tukey HSD analyses were performed following ANOVA to assess mean differences between groups. Means with the same superscript letter (a, b, c) are not significantly different from each other, while means with different letters indicate significant differences, according to Tukey’s post hoc test (*p* < 0.05). ^2^ “Total” refers to the mean (SD) obtained from the one-way ANOVA analysis for each measurement across all individuals in the respective brain region, regardless of the oil received. SOD, Superoxide Dismutase; CAT, Catalase; GPx, Glutathione Peroxidase; GR, Glutathione Reductase; GSH, Reduced Glutathione; GSSG, Oxidized Glutathione.

**Table 3:** Influence of the degree of unsaturation of fatty acids in oils administered to the rats on lipid peroxidation products and protein oxidation in rat cortex and cerebellum ^1^.

	Region	Total ^2^	Coconut	Soybean	EPA/DHA 1:1	80% DHA
CD (mmoles hydroperoxide/kg lipid)	Cortex	5.72 (0.38)	5.19 (0.78)	5.88 (0.55)	5.74 (0.92)	6.08 (0.54)
	Cerebellum	5.97 (0.84)	5.52 (0.27)	5.09 (0.26)	6.25 (1.41)	7.00 (1.59)
MDA (a.u./µg lipid) *	Cortex	138.33 (2.89)	137.37 (9.98)	135.15 (5.19)	142.05 (10.33)	138.74 (19.58)
	Cerebellum	107.72 * (3.78)	106.10 (4.32)	112.04 (3.60)	105.02 (5.39)	102.78 (5.01)
HNE (a.u./µg lipid) *	Cortex	136.61 (3.35)	135.24 (6.42)	132.76 (4.52)	140.51 (7.00)	137.93 (24.63)
	Cerebellum	115.00 * (3.35)	119.97 (10.23)	113.93 (3.77)	113.40 (4.77)	112.69 (5.70)
HHE (a.u./µg lipid) *	Cortex	263.61 (7.62)	266.06 (16.69)	252.42 (19.80)	266.42 (16.72)	269.52 (64.00)
	Cerebellum	210.06 * (4.98)	217.38 (5.22)	208.99 (0.79)	206.63 (6.42)	207.23 (9.90)
oxPC (a.u./µg lipid) *	Cortex	617.99 (64.62)	626.12 (59.09)	590.50 (44.01)	625.32 (42.97)	630.04 (112.42)
	Cerebellum	544.60 * (29.55)	524.79 (29.51)	535.05 (25.62)	544.41 (41.10)	574.15 (21.95)
oxPC (a.u./µg PC) *	Cortex	456.37 (62.97)	483.05 (71.97)	498.31 (50.01)	438.73 (35.63)	405.39 (94.26)
	Cerebellum	445.24 * (30.89)	454.25 (45.91)	506.01 (31.71)	422.02 (25.84)	398.69 (20.11)
MDA-protein adducts (a.u./µg protein) *	Cortex	1.20 (0.31)	1.19 (0.30)	1.23 (0.39)	1.32 (0.31)	1.05 (0.24)
	Cerebellum	1.03 * (0.21)	1.01 (0.20)	1.06 (0.18)	0.97 (0.16)	1.10 (0.32)
HNE-protein adducts (a.u./µg protein) *	Cortex	1.91 (0.65)	1.75 (0.64)	1.54 (0.44)	2.01 (0.80)	2.32 (0.51)
	Cerebellum	0.80 * (0.16)	0.79 (0.11)	0.75 (0.14)	0.68 (0.15)	0.72 (0.15)
HHE-protein adducts (a.u./µg protein) *	Cortex	1.10 (0.25)	1.13 (0.22)	0.98 (0.15)	1.10 (0.28)	1.20 (0.33)
	Cerebellum	1.12 (0.41)	1.17 (0.29)	0.98 (0.20)	1.30 (0.70)	1.03 (0.32)
oxPC-protein adducts (a.u./µg protein) *	Cortex	1.05 (0.18)	1.07 (0.23)	1.11 (0.20)	1.05 (0.14)	0.97 (0.13)
	Cerebellum	0.95 * (0.16)	0.85 (0.02)	0.92 (0.15)	1.12 (0.14)	0.91 (0.12)
Total protein carbonylation (a.u./µg protein) *^$^	Cortex	2.94 (0.44)	3.18 ^a^ (0.39)	3.05 ^a^ (0.05)	3.10 ^a^ (0.41)	2.41 ^b&^ (0.40)
	Cerebellum	1.30 * (0.17)	1.23 ^c^ (0.16)	1.34 ^c^ (0.07)	1.28 ^c^ (0.30)	1.37 ^c^ (0.16)
Cytosolic protein carbonylation (a.u./µg protein) *	Cortex	0.43 (0.07)	0.47 (0.07)	0.39 (0.08)	0.39 (0.01)	0.46 (0.07)
	Cerebellum	0.22 * (0.02)	0.23 (0.02)	0.21 (0.03)	0.21 (0.01)	0.21 (0.02)
Myofibrillar protein carbonylation (a.u./µg protein) *^$^	Cortex	2.51 (0.47)	2.71 ^a^ (0.46)	2.66 ^a^ (0.05)	2.71 ^a^ (0.41)	1.95 ^b&^ (0.47)
	Cerebellum	1.09 * (0.18)	1.00 ^c^ (0.16)	1.13 ^c^ (0.10)	1.07 ^c^ (0.29)	1.16 ^c^ (0.17)
Protein-SSG (a.u./µg protein) *	Cortex	0.50 (0.02)	0.52 (0.08)	0.49 (0.08)	0.49 (0.11)	0.49 (0.09)
	Cerebellum	0.16 * (0.02)	0.17 (0.05)	0.17 (0.03)	0.14 (0.03)	0.14 (0.04)

^1^ Values are expressed as mean (standard deviation) (*n* = 10). Two-way ANOVA analyses were conducted. * *p* < 0.05 significant differences due to the factor “tissue” (Cortex and Cerebellum); ^$^ *p* < 0.05 significant differences due to the factor “oil” (Coconut, Soybean, EPA/DHA 1:1 and 80% DHA). Post hoc Tukey HSD analyses were performed following ANOVA to assess mean differences between groups. Means with the same superscript letter (a, b, c) are not significantly different from each other, while means with different letters indicate significant differences, according to Tukey’s post hoc test (*p* < 0.05). ^&^ *p* < 0.1. ^2^ “Total” refers to the mean (SD) obtained from the two-way ANOVA analysis for each measurement across all individuals in the respective brain region, regardless of the oil received. CD, Conjugated Dienes; MDA, Malondialdehyde; HNE, 4-hydroxynonenal; HHE, Hydroxyhexenal; oxPC, Oxidized Phosphatidylcholines; a.u., Arbitrary Units.

**Table 4:** Proteins found carbonylated in both the cortex and the cerebellum of 8-month-old Sprague Dawley rats ^1^.

Protein Name	Gene Name	Uniprot Code	Region	Relative Protein Amount (a.u.) ^2^	Protein Carbonylation Index
Creatine kinase B-type OS = *Rattus norvegicus* OX = 10116 GN = Ckb PE = 1 SV = 2	*Ckb*	P07335	Cortex	11.17 (1.18)	1.18 (0.20)
			Cerebellum	6.40 * (0.90)	0.46 * (0.13)
ATP synthase subunit beta mitochondrial OS = *Rattus norvegicus* OX = 10116 GN = Atp5f1b PE = 1 SV = 2	*Atp5f1b*	P10719	Cortex	0.60 (0.12)	0.65 (0.14)
			Cerebellum	3.97 * (0.55)	1.08 * (0.19)
Pyruvate kinase PKM OS = *Rattus norvegicus* OX = 10116 GN = Pkm PE = 1 SV = 3	*Pkm*	P11980	Cortex	1.35 (0.59)	0.45 (0.24)
			Cerebellum	1.42 * (0.32)	0.94 * (0.51)
Pyruvate dehydrogenase E1 component subunit alpha somatic form mitochondrial OS = *Rattus norvegicus* OX = 10116 GN = Pdha1 PE = 1 SV = 2	*Pdha1*	P26284	Cortex	0.76 (0.13)	0.93 (0.19)
			Cerebellum	1.97 * (0.32)	1.11 (0.18)
Pyruvate dehydrogenase E1 component subunit beta mitochondrial OS = *Rattus norvegicus* OX = 10116 GN = Pdhb PE = 1 SV = 2	*Pdhb*	P49432	Cortex	0.42 (0.07)	1.15 (0.34)
			Cerebellum	0.59 * (0.08)	1.34 (0.33)
Aconitate hydratase mitochondrial OS = *Rattus norvegicus* OX = 10116 GN = Aco2 PE = 1 SV = 2	*Aco2*	Q9ER34	Cortex	0.52 (0.13)	0.67 (0.15)
			Cerebellum	1.33 * (0.29)	1.03 * (0.27)
L-lactate dehydrogenase B chain OS = *Rattus norvegicus* OX = 10116 GN = Ldhb PE = 1 SV = 2	*Ldhb*	P42123	Cortex	0.72 (0.13)	0.84 (0.21)
			Cerebellum	2.58 * (0.29)	0.57 * (0.15)
Fructose-bisphosphate aldolase A OS = *Rattus norvegicus* OX = 10116 GN = Aldoa PE = 1 SV = 2	*Aldoa*	P05065	Cortex	1.18 (0.26)	1.30 (0.31)
			Cerebellum	0.57 * (0.14)	1.53 (0.78)
Fructose-bisphosphate aldolase C OS = *Rattus norvegicus* OX = 10116 GN = Aldoc PE = 1 SV = 3	*Aldoc*	P09117	Cortex	2.96 (0.35)	1.08 (0.22)
			Cerebellum	3.32 (0.41)	1.22 (0.16)
Triosephosphate isomerase OS = *Rattus norvegicus* OX = 10116 GN = Tpi1 PE = 1 SV = 2	*Tpi1*	P48500	Cortex	1.38 (0.15)	2.78 (0.41)
			Cerebellum	1.20 (0.25)	1.61 * (0.45)
Glyceraldehyde-3-phosphate dehydrogenase OS = *Rattus norvegicus* OX = 10116 GN = Gapdh PE = 1 SV = 3	*Gapdh*	P04797	Cortex	2.38 (0.48)	0.96 (0.17)
			Cerebellum	2.94 (0.78)	1.21 (0.41)
Alpha-enolase OS = *Rattus norvegicus* OX = 10116 GN = Eno1 PE = 1 SV = 4	*Eno1*	P04764	Cortex	1.77 (0.48)	0.58 (0.13)
			Cerebellum	2.39 * (0.44)	0.64 (0.14)
Gamma-enolase OS = *Rattus norvegicus* OX = 10116 GN = Eno2 PE = 1 SV = 2	*Eno2*	P07323	Cortex	1.39 (0.34)	0.45 (0.10)
			Cerebellum	1.37 (0.24)	0.53 (0.19)
Aspartate aminotransferase cytoplasmic OS = *Rattus norvegicus* OX = 10116 GN = Got1 PE = 1 SV = 3	*Got1*	P13221	Cortex	0.39 (0.07)	0.84 (0.20)
			Cerebellum	0.90 * (0.21)	1.43 * (0.26)
Glutamine synthetase OS = *Rattus norvegicus* OX = 10116 GN = Glul PE = 1 SV = 3	*Glul*	P09606	Cortex	2.66 (0.36)	0.83 (0.13)
			Cerebellum	2.63 (0.33)	1.12 * (0.14)
Glutamate dehydrogenase 1 mitochondrial OS = *Rattus norvegicus* OX = 10116 GN = Glud1 PE = 1 SV = 2	*Glud1*	P10860	Cortex	0.54 (0.08)	0.89 (0.31)
			Cerebellum	3.76 * (0.62)	0.58 * (0.10)
Actin cytoplasmic 1 OS = *Rattus norvegicus* OX = 10116 GN = Actb PE = 1 SV = 1/Actin cytoplasmic 2 OS = *Rattus norvegicus* OX = 10116 GN = Actg1 PE = 1 SV = 1	*Actb/Actg1*	P60711/P63259	Cortex	3.83 (0.85)	0.86 (0.25)
			Cerebellum	8.89 * (1.33)	1.14 * (0.19)
Tubulin beta-2A chain OS = *Rattus norvegicus* OX = 10116 GN = Tubb2a PE = 1 SV = 1	*Tubb2a*	P85108	Cortex	3.72 (1.07)	1.40 (0.27)
			Cerebellum	1.66 * (0.55)	0.61 * (0.17)
Septin-11 OS = *Rattus norvegicus* OX = 10116 GN = Septin11 PE = 1 SV = 1	*Septin11*	B3GNI6	Cortex	0.51 (0.07)	0.63 (0.11)
			Cerebellum	0.45 (0.06)	0.87 * (0.17)
Syntaxin-binding protein 1 OS = *Rattus norvegicus* OX = 10116 GN = Stxbp1 PE = 1 SV = 1	*Stxbp1*	P61765	Cortex	1.09 (0.20)	0.44 (0.09)
			Cerebellum	0.56 * (0.11)	0.89 * (0.26)
Synapsin-1 OS = *Rattus norvegicus* OX = 10116 GN = Syn1 PE = 1 SV = 3	*Syn1*	P09951	Cortex	1.79 (0.54)	1.91 (0.65)
			Cerebellum	0.70 * (0.19)	1.86 (0.77)
Dihydropyrimidinase-related protein 2 OS = *Rattus norvegicus* OX = 10116 GN = Dpysl2 PE = 1 SV = 1	*Dpysl2*	P47942	Cortex	2.93 (0.35)	0.66 (0.07)
			Cerebellum	1.60 * (0.16)	0.82 (0.30)
Guanine nucleotide-binding protein G(o) subunit alpha OS = *Rattus norvegicus* OX = 10116 GN = Gnao1 PE = 1 SV = 2	*Gnao1*	P59215	Cortex	0.53 (0.11)	0.76 (0.21)
			Cerebellum	0.44 (0.10)	0.84 (0.25)
Heat shock cognate 71 kDa protein OS = *Rattus norvegicus* OX = 10116 GN = Hspa8 PE = 1 SV = 1	*Hspa8*	P63018	Cortex	1.92 (0.44)	0.38 (0.09)
			Cerebellum	3.34 * (0.94)	0.40 (0.12)
60 kDa heat shock protein mitochondrial OS = *Rattus norvegicus* OX = 10116 GN = Hspd1 PE = 1 SV = 1	*Hspd1*	P63039	Cortex	0.50 (0.07)	0.67 (0.14)
			Cerebellum	0.97 * (0.13)	0.73 (0.16)
Albumin OS = *Rattus norvegicus* OX = 10116 GN = Alb PE = 1 SV = 2	*Alb*	P02770	Cortex	1.14 (0.13)	0.89 (0.13)
			Cerebellum	0.46 * (0.09)	1.18 (0.39)
Ubiquitin carboxyl-terminal hydrolase isozyme L1 OS = *Rattus norvegicus* OX = 10116 GN = Uchl1 PE = 1 SV = 2	*Uchl1*	Q00981	Cortex	0.80 (0.08)	1.19 (0.24)
			Cerebellum	<0.001 *	<0.001 *

^1^ Values are expressed as mean (standard deviation) (*n* = 40). Student’s *t*-tests were conducted to assess mean differences in protein quantity and carbonylation index of common carbonylated proteins between the cortex and cerebellum. Differences were considered statistically significant at * *p* < 0.05. ^2^ Coomassie intensity in arbitrary units (a.u.).

## Data Availability

The data presented in this study are available in the article and Supplementary Materials.

## Supplementary Materials

The following supporting information can be downloaded at: https://www.mdpi.com/article/10.3390/antiox13111408/s1, Figure S1. Representative 2-DE gel images showing carbonylated and total proteins identified in the cytosolic fraction of rat cortex. Upper panels are the FTSC-stained 2-DE gel images, and bottom panels are the corresponding Coomassie-stained 2-DE gel images from (a) Coconut oil, (b) Soybean oil, (c) EPA/DHA 1:1 oil and (d) 80%DHA oil experimental groups. Numbered protein spots (1–43) indicate the carbonylated proteins confidently identified and listed in Supplementary Table S4. Images are representative of three independent labeling experiments performed in triplicate; Figure S2. Representative 2-DE gel images showing carbonylated and total proteins identified in the myofibrillar fraction of rat cortex. Upper panels are the FTSC-stained 2-DE gel images, and bottom panels are the corresponding Coomassie-stained 2-DE gel images from (a) Coconut oil, (b) Soybean oil, (c) EPA/DHA 1:1 oil and (d) 80%DHA oil experimental groups. Numbered protein spots (44–77) indicate the carbonylated proteins confidently identified and listed in Supplementary Table S4. Images are representative of three independent labeling experiments performed in triplicate; Figure S3. Representative 2-DE gel images showing carbonylated and total proteins identified in the cytosolic fraction of rat cerebellum. Upper panels are the FTSC-stained 2-DE gel images, and bottom panels are the corresponding Coomassie-stained 2-DE gel images from (a) Coconut oil, (b) Soybean oil, (c) EPA/DHA 1:1 oil and (d) 80%DHA oil experimental groups. Numbered protein spots (1–41) indicate the carbonylated proteins confidently identified and listed in Supplementary Table S4. Images are representative of three independent labeling experiments performed in triplicate; Figure S4. Representative 2-DE gel images showing carbonylated and total proteins identified in the myofibrillar fraction of rat cerebellum. Upper panels are the FTSC-stained 2-DE gel images, and bottom panels are the corresponding Coomassie-stained 2-DE gel images from (a) Coconut oil, (b) Soybean oil, (c) EPA/DHA 1:1 oil and (d) 80%DHA oil experimental groups. Numbered protein spots (42–76) indicate the carbonylated proteins confidently identified and listed in Supplementary Table S4. Images are representative of three independent labeling experiments performed in triplicate; Table S1. Diet composition; Table S2: Fatty acid composition of supplemented oils (mg/100 mg); Table S3: Biometrical and biochemical data of Sprague Dawley rats fed a standard diet supplemented with coconut oil, soybean oil, EPA/DHA 1:1 oil and 80%DHA oil; Table S4. Carbonylated protein identified in the cortex. Nº Spot column numbers refer to the numbered spots in Figures S1–S4 (pointed out in the column “Figure”). One-way ANOVA analyses were conducted. * *p* < 0.05 significant differences given by the factor “supplement” (COCONUT, SOYBEAN, EPA/DHA 1:1 and 80% DHA). Means with different superscript letters indicate significant differences (*p* < 0.05) (analyzed by post hoc Tukey HSD test). References [24,29,64] are cited in the supplementary materials.
