# Supplementary material for: Influence of the Degree of Unsaturation in Fish Oil Supplements on Oxidative Stress and Protein Carbonylation in the Cerebral Cortex and Cerebellum of Healthy Rats
Source: Antioxidants (Basel). 2024 Nov 17;13(11):1408. doi: 10.3390/antiox13111408 (PMC11591239; doi:10.3390/antiox13111408)
Supplement: Supplementary file 1 [file antioxidants-13-01408-s001.zip › Supplementary Figures S1-S4.pdf]

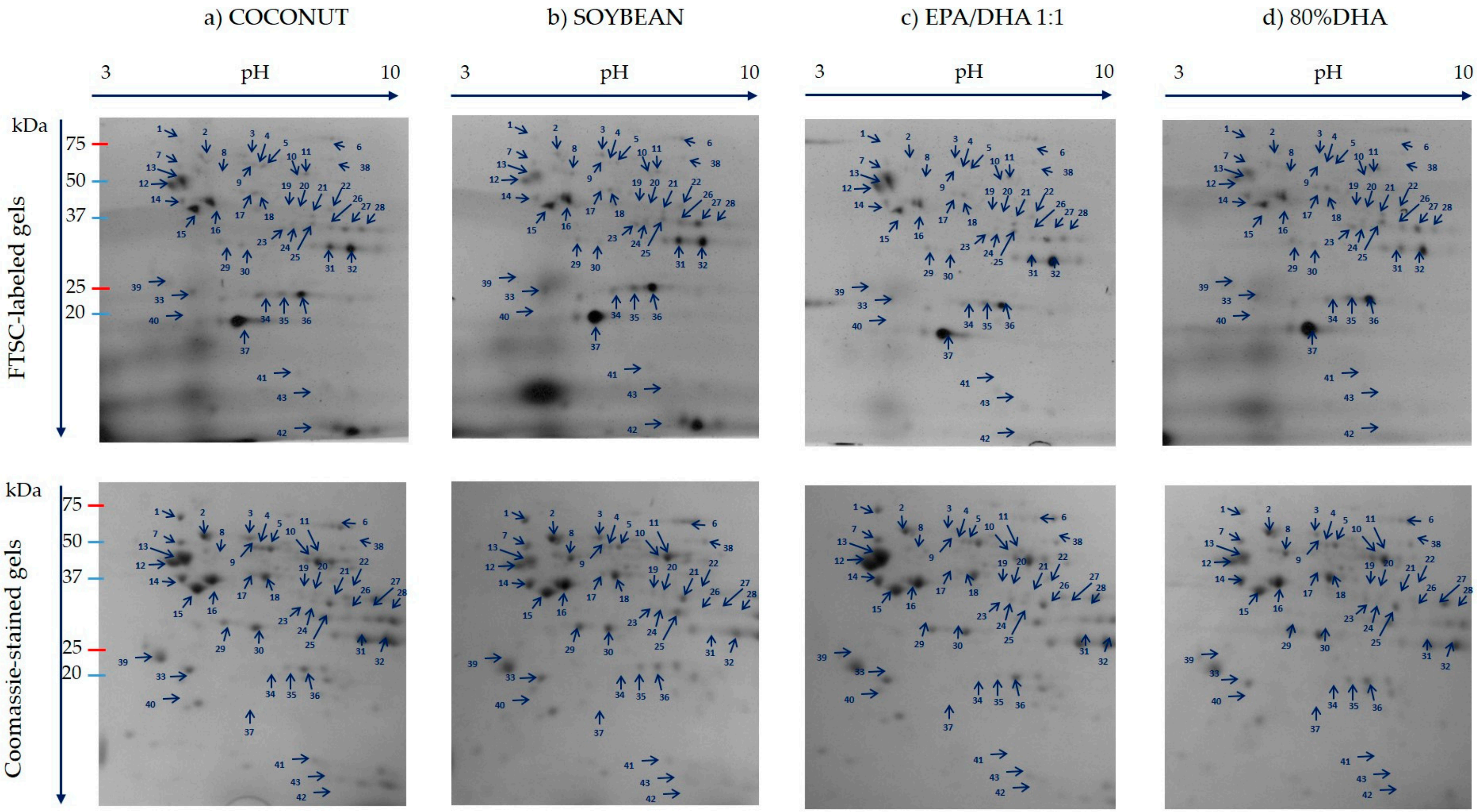

Figure S1. Representative 2-DE gel images showing carbonylated and total proteins identified in the cytosolic fraction of rat cortex. Upper panels are the FTSC-stained 2-DE gel images and bottom panels are the corresponding Coomassie-stained 2-DE gel images from a) Coconut oil, b) Soybean oil, c) EPA/DHA 1:1 oil and d) 80%DHA oil experimental groups. Numbered protein spots (1-43) indicate carbonylated proteins confidently identified and listed in Supplementary Table S4. Images are representatives of three independent labelling experiments performed in triplicates.

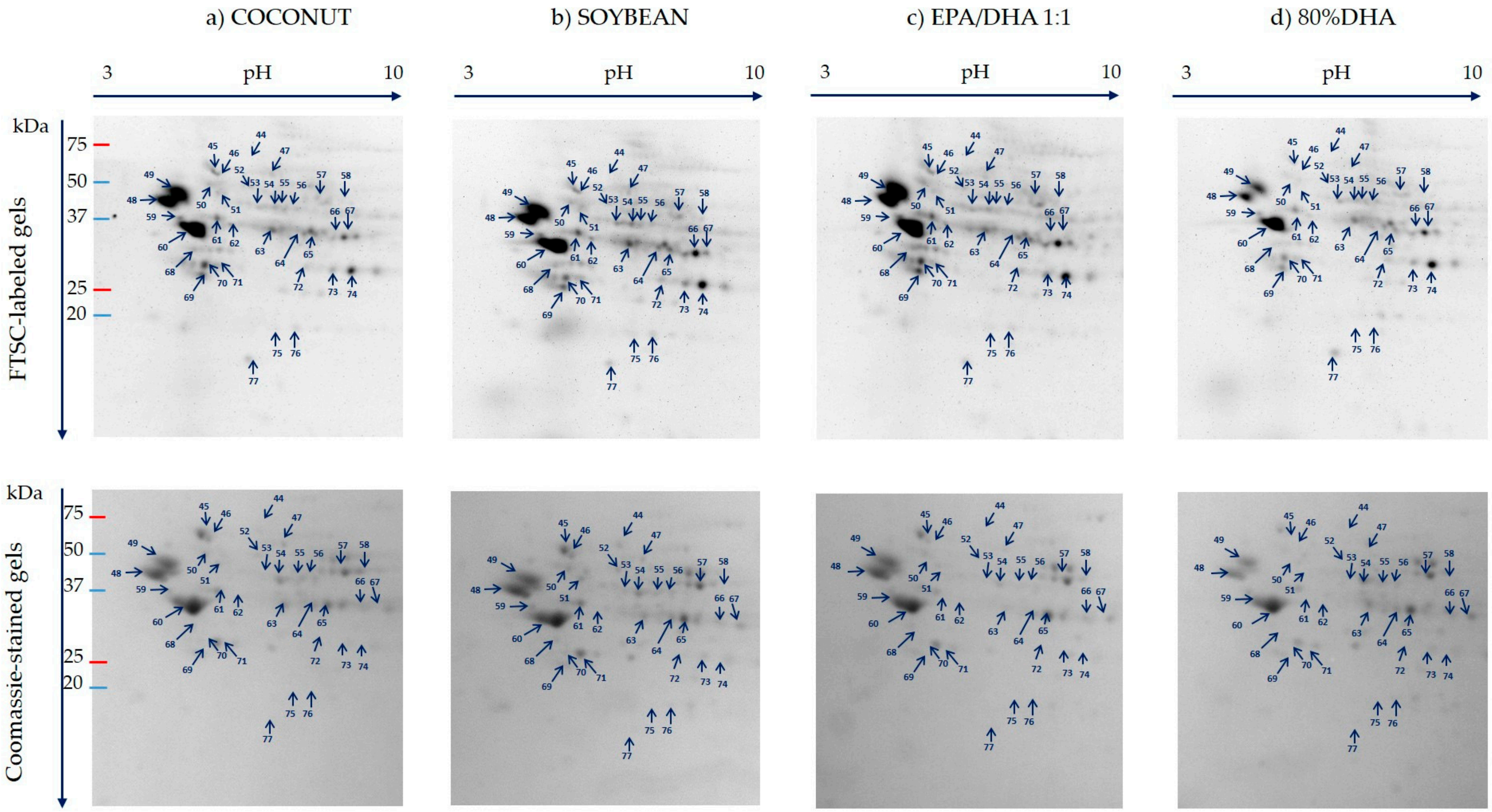

Figure S2. Representative 2-DE gel images showing carbonylated and total proteins identified in the myofibrillar fraction of rat cortex. Upper panels are the FTSC-stained 2-DE gel images and bottom panels are the corresponding Coomassie-stained 2-DE gel images from a) Coconut oil, b) Soybean oil, c) EPA/DHA 1:1 oil and d) 80%DHA oil experimental groups. Numbered protein spots (44-77) indicate carbonylated proteins confidently identified and listed in Supplementary Table S4. Images are representatives of three independent labelling experiments performed in triplicates.

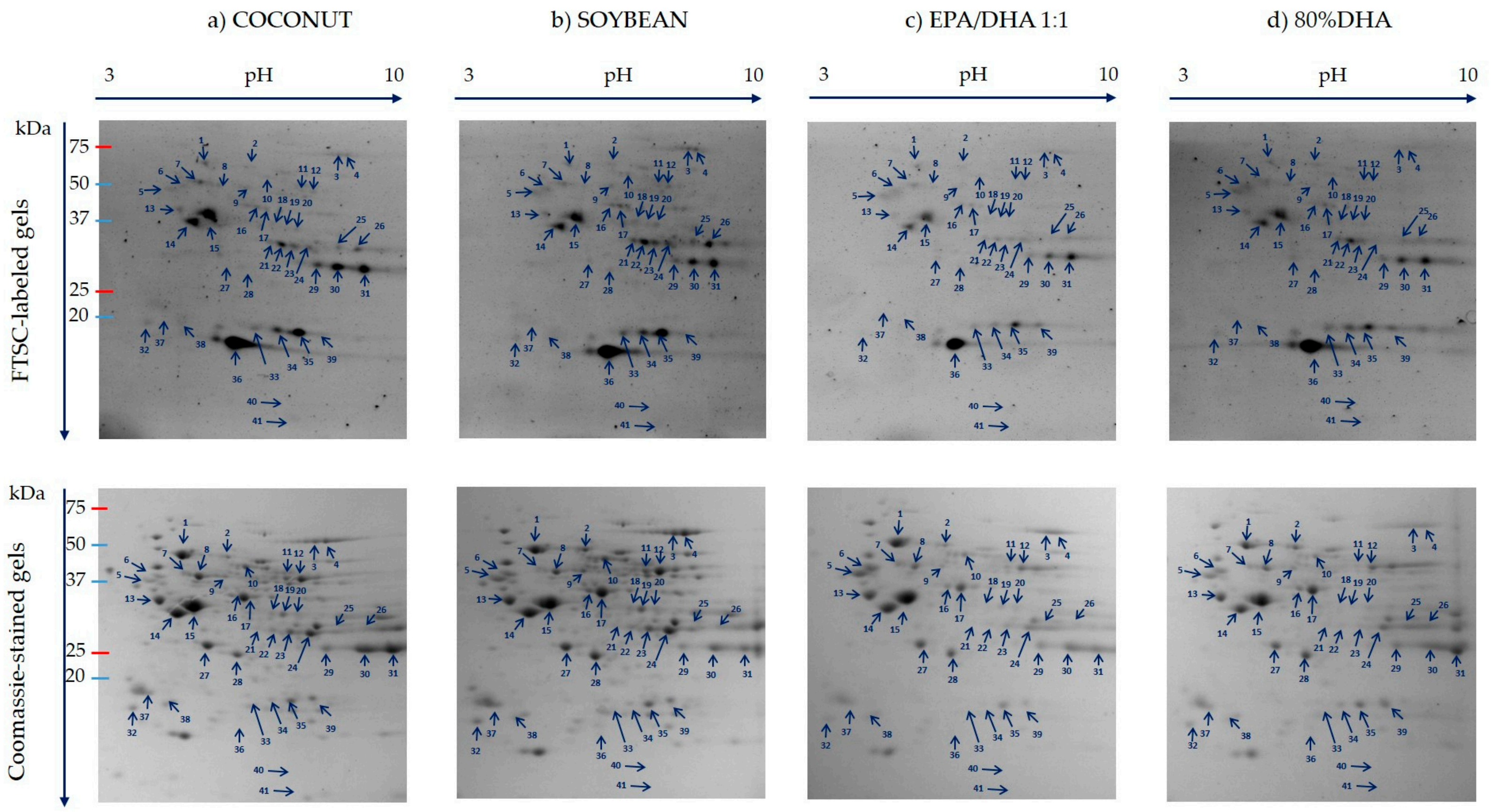

Figure S3. Representative 2-DE gel images showing carbonylated and total proteins identified in the cytosolic fraction of rat cerebellum. Upper panels are the FTSC-stained 2-DE gel images and bottom panels are the corresponding Coomassie-stained 2-DE gel images from a) Coconut oil, b) Soybean oil, c) EPA/DHA 1:1 oil and d) 80%DHA oil experimental groups. Numbered protein spots (1-41) indicate carbonylated proteins confidently identified and listed in Supplementary Table S4. Images are representatives of three independent labelling experiments performed in triplicates.

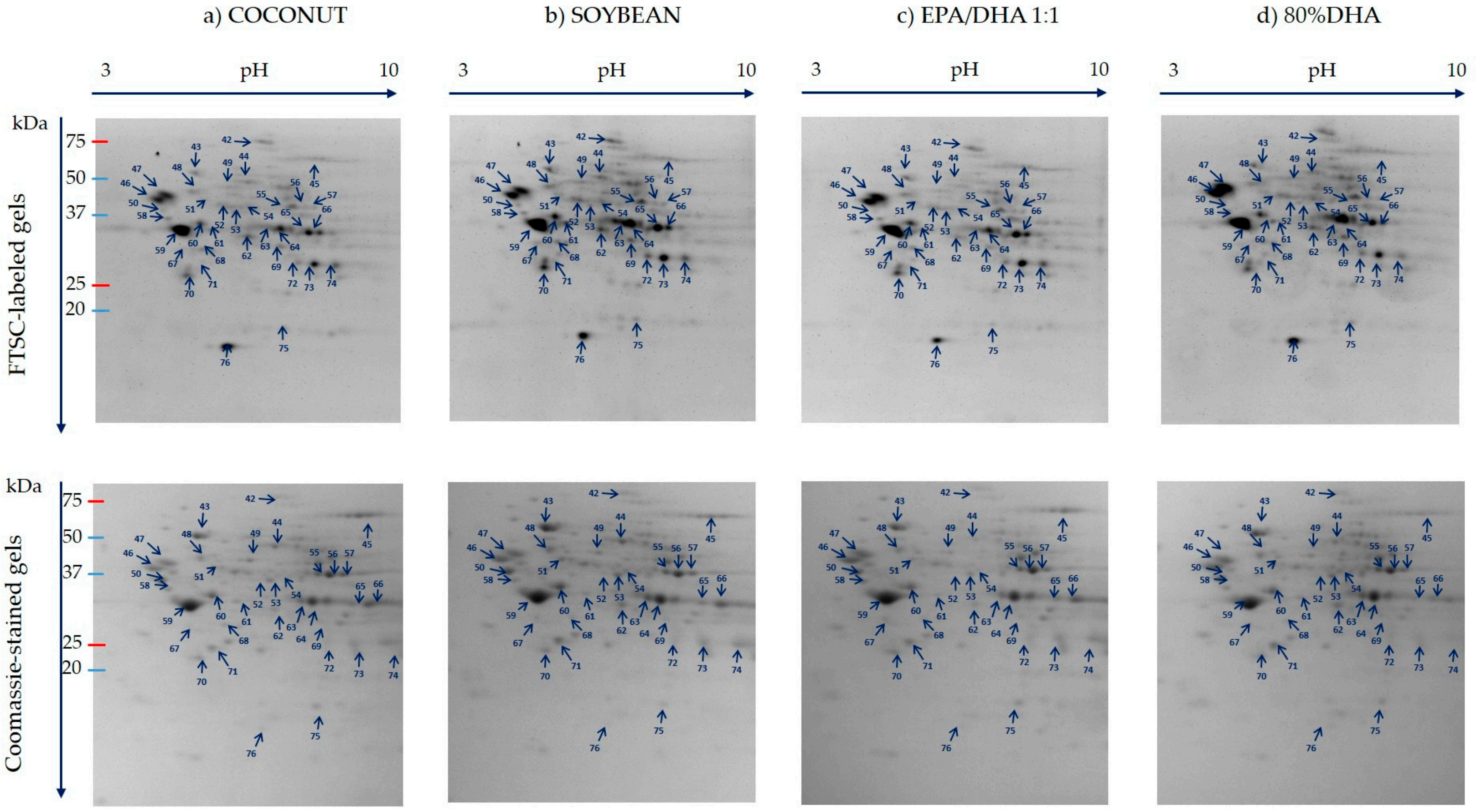

Figure S4. Representative 2-DE gel images showing carbonylated and total proteins identified in the myofibrillar fraction of rat cerebellum. Upper panels are the FTSC-stained 2-DE gel images and bottom panels are the corresponding Coomassie-stained 2-DE gel images from a) Coconut oil, b) Soybean oil, c) EPA/DHA 1:1 oil and d) 80%DHA oil experimental groups. Numbered protein spots (42-76) indicate carbonylated proteins confidently identified and listed in Supplementary Table S4. Images are representatives of three independent labelling experiments performed in triplicates.
