# Supplementary material for: Influence of the Degree of Unsaturation in Fish Oil Supplements on Oxidative Stress and Protein Carbonylation in the Cerebral Cortex and Cerebellum of Healthy Rats
Source: Antioxidants (Basel). 2024 Nov 17;13(11):1408. doi: 10.3390/antiox13111408 (PMC11591239; doi:10.3390/antiox13111408)
Supplement: Supplementary file 1 [file antioxidants-13-01408-s001.zip › Supplementary Tables S1-S3.pdf]

Table S1. Diet composition.*

	Composition (All groups)
**Crude protein (%)**	14.3
**Fat (%)**	4.0
**Carbohydrates (%)**	48.0
**Crude fiber (%)**	4.1
**Neutral detergent fiber (%)**	18.0
**Ash (%)**	4.7
**Energy density (kcal/g)**	2.9
**Calories from protein (%)**	20
**Calories from fat (%)**	13
**Calories from carbohydrates (%)**	67

*The composition of diet described by the manufacturer was used (Teklad Global 14%; Envigo, Indianápolis, IN, EE.UU.)

Table S2: Fatty acid composition of supplemented oils (mg/100mg).*

Fatty acid (%)	Coconut	Soy	EPA/DHA 1:1	80%DHA
6:0	0.2–0.5	-	-	-
8:0	5.4–9.5	-	-	-
10:0	4.5–9.7	-	-	-
12:0	44.1–51.0	-	-	-
14:0	13.1-18.5	0.12 (0.03)	1.40 (0.05)	ND
15:0	-	ND	0.43 (0.06)	ND
16:0	7.5-10.5	10.93 (0.13)	8.39 (0.04)	0.12 (0.02)
16:1ω7	-	0.10 (0.00)	1.92 (0.00)	0.26 (0.01)
17:0	-	0.16 (0.01)	1.38 (0.01)	0.16 (0.02)
18:0	1.0–3.2	4.14 (0.08)	3.74 (0.01)	0.11 (0.02)
18:1ω9	5.0–8.2	23.30 (0.18)	8.09 (0.02)	0.23 (0.03)
18:1ω7	-	2.11 (0.01)	1.88 (0.02)	ND
18:2ω6	1.0–2.6	50.75 (0.36)	1.29 (0.01)	0.09 (0.02)
18:3ω3	-	7.02 (0.02)	0.45 (0.03)	ND
20:0	0.2–1.5	0.33 (0.01)	0.47 (0.00)	ND
20:1ω9	-	0.20 (0.02)	2.44 (0.01)	ND
20:2ω6	-	ND	0.43 (0.02)	ND
20:3ω6	-	ND	0.32 (0.01)	ND
20:4ω3	-	ND	1.46 (0.03)	0.26 (0.04)
20:4ω6	-	0.49 (0.04)	2.65 (0.04)	0.75 (0.02)
20:5ω3	-	0.10 (0.01)	27.56 (0.04)	3.87 (0.20)
22:4ω6	-	0.25 (0.02)	1.82 (0.00)	1.15 (0.02)
22:5ω3	-	ND	3.16 (0.01)	4.09 (0.01)
22:5ω6	-	ND	1.52 (0.10)	6.18 (0.40)
22:6ω3	-	ND	26.15 (0.03)	82.63 (0.15)
24:1ω9	-	ND	0.43(0.01)	ND

* Fatty acid composition of the soybean and the fish oils was measured according to the method described by Lepage and Roy [29], whereas fatty acid composition of coconut oil described by Lal et al.[64] was used. Abbreviations: ND: non-detected.

29. Lepage, G.; Roy, C.C. Direct Transesterification of All Classes of Lipids in a One-Step Reaction. *J. Lipid Res.* **1986**, *27*, 114–120. https://doi.org/10.1016/S0022-2275(20)38861-1.

64 Lal, J., Kumar, S. & Madambath, I. Coconut Palm Coconut Palm. In *Encyclopedia of Food Sciences and Nutrition*; Academic Press: London, UK, 2003; pp. 1464–1475. https://doi.org/doi:10.1016/B0-12-227055-X/00263-7.

Table S3: Biometrical and biochemical data from Sprague-Dawley rats fed a standard diet supplemented[1,2] with Coconut oil, Soy oil, EPA/DHA 1:1 oil and 80%DHA oil.

Biochemical parameters	Coconut Mean (SD)	Soy Mean (SD)	EPA/DHA (1:1) Mean (SD)	80%DHA Mean (SD)
**Initial body weight (g)**	403[a] (22)	401[a] (18)	399[a] (15)	398[a] (14)
**Final body weight (g)**	435[a] (29)	442[a] (21)	435[a] (15)	441[a] (25)
**Perigonadal adipose tissue weigh (g)**	5.0[a] (1.4)	5.2[a] (1.1)	4.7[a] (0.7)	4.8[a] (1.0)
**Adiposity index (%)[y]**	1.2[a] (0.2)	1.2[a] (0.2)	1.1[a] (0.1)	1.1[a] (0.2)
**Blood glucose (mmol/L)***	5.1[a] (0.3)	5.5[b] (0.4)	5.0[a] (0.2)	5.0[a] (0.2)
**Blood HbA1c (%)**	2.9[a] (0.1)	3.0[a] (0.3)	3.2[a] (0.2)	3.2[a] (0.3)
**Plasma Fat content (%)***	0.52[a] (0.04)	0.45[ab] (0.03)	0.44[bc] (0.03)	0.37[c] (0.04)
**Plasma TAG (mmol/L)**	0.7[a] (0.1)	0.7[a] (0.2)	0.7[a] (0.2)	0.7[a] (0.2)
**Plasma TC (mmol/L)***	2.7[a] (0.3)	2.5[a] (0.4)	2.4[ab] (0.2)	2.1[b] (0.2)
**Plasma HDL (mmol/L)***	1.80[a] (0.21)	1.69[ab] (0.21)	1.62[b] (0.12)	1.47[c] (0.15)
**Plasma LDL (mmol/L)***	0.45[a] (0.08)	0.35[b] (0.13)	0.45[a] (0.05)	0.39[ab] (0.09)
**Plasma LDL/HDL ratio***	0.25[a] (0.03)	0.20[b] (0.06)	0.28[c] (0.03)	0.27[ac] (0.06)
**Liver fat content (%)***	5.36[a] (0.19)	5.13[ab] (0.35)	4.92[b] (0.31)	5.12[a] (0.32)
**Liver TAG (µmol/g tissue)**	11.2[a] (1.1)	10.9[a] (1.7)	11.0[a] (1.3)	10.9[a] (1.3)
**Liver TC (µmol/g tissue)**	4.7[a] (0.9)	4.8[a] (1.1)	5.8[a] (0.6)	5.6[a] (0.6)
**Muscle fat content (%)***	2.13[a] (0.67)	2.27[a] (0.53)	2.17[ab] (0.43)	1.64[b] (0.17)
**Plasma AST (U/L)**	91.2[a] (29.4)	85.8[a] (31.7)	110.2[a] (15.5)	120.7[a] (21.0)
**Plasma ALT (U/L)**	43.9[a] (15.9)	43.8[a] (11.3)	49.0[a] (12.2)	53.8[a] (23.8)
**Plasma AST/ALT ratio**	2.13[a] (0.55)	1.92[a] (0.34)	2.39[a] (0.71)	2.49[a] (0.88)
**Fat content (erythrocite) (%)**	2.05[a] (0.19)	2.04[a] (0.20)	1.98[a] (0.26)	1.98[a] (0.23)

[1] One-way ANOVA analyses were conducted. * $p<0.05$ significant differences given by the factor "supplement" (Coconut, Soy, EPA/DHA 1:1, 80%DHA). Means with different superscript indicate significant differences between groups (*p<0.05*) (analyzed by post-hoc Scheffé and Mann-Whitney). [y] Adiposity index: (total abdominal fat×100)/body weight. HbA1c: Glycated hemoglobin; TAG: Triacylglicerol; TC: Total cholesterol; HDL: High density lipoprotein; LDL: Low density lipoprotein; AST: Aspartate aminotranferase; ALT: Alanine aminotransferase

[2] Parameters in the table are already published in: 24. Miralles-Pérez, B.; Méndez, L.; Nogués, M.R.; Sánchez-Martos, V.; Fortuño-Mar, À.; Ramos-Romero, S.; Hereu, M.; Medina, I.; Romeu, M. Effects of a Fish Oil Rich in Docosahexaenoic Acid on Cardiometabolic Risk Factors and Oxidative Stress in Healthy Rats. *Mar. Drugs* **2021**, *19*, 555. https://doi.org/10.3390/md19100555.
